# Management of Pelvic Ring Injury Patients With Hemodynamic Instability

**DOI:** 10.3389/fsurg.2020.588845

**Published:** 2020-11-12

**Authors:** Meir Marmor, Ashraf N. El Naga, Jordan Barker, Jacob Matz, Styliani Stergiadou, Theodore Miclau

**Affiliations:** ^1^Department of Orthopedic Surgery, University of California, San Francisco, San Francisco, CA, United States; ^2^Faculty of Medicine, University of Thessaly, Larissa, Greece

**Keywords:** pelvic fracture, hemodynamic instability, angioembolization, pre-peritoneal pelvic packing, bleeding, triage, resuscitation, shock

## Abstract

Pelvic ring injuries (PRI) are among the most difficult injuries to deal with in orthopedic trauma. When these injuries are accompanied by hemodynamic instability their management becomes significantly more complex. A methodical assessment and expeditious triage are required for these patients followed by adequate resuscitation. A major triage decision is whether these patients should undergo arterial embolization in the angiography suit or prompt packing and pelvic stabilization in the operating room. Patient characteristics, fracture type and injury characteristics are taken into consideration in the decision-making process. In this review we discuss the acute evaluation, triage and management of PRIs associated with hemodynamic instability. An evidence based and protocol driven approach is necessary in order to achieve optimal outcomes in these patients.

## Introduction

Pelvic ring injuries (PRIs) with hemodynamic instability typically occur in polytrauma patients. The initial assessment of the polytrauma patient follows the Advanced Trauma Life Support (ATLS) guidelines (1), which call for evaluation and treatment of airway and breathing problems first and then assessment and treatment of hemodynamic instability (HI). The ATLS guidelines define HI as having a blood pressure <90 mmHg and heart rate >120 bpm, with evidence of skin vasoconstriction (cool, clammy, and decreased capillary refill), altered level of consciousness, and/or shortness of breath (1). Other definitions include having a systolic blood pressure (SBP) of >90 mmHg but requiring at least 4–6 units of packed red blood cells within the first 24 h, vasopressor drugs (2, 3), and/or have an admission base deficit (BD) >6 mmol/L and/or a shock index (heart rate divided by SBP) >1 (4, 5). In the setting of trauma, HI is always assumed to be the result of active bleeding or significant blood loss prior to hospital arrival. The pelvic ring, if broken, can be a major contributor to HI and therefore the expeditious evaluation and treatment of pelvic bleeding is a crucial part of managing trauma patients with HI.

## Initial Assessment and Triage

### Physical and Laboratory Examination

HI is diagnosed during the initial physical examination, also known as the ATLS primary survey, which also attempts to identify whether significant bleeding exists in the head, chest, abdomen, or extremities (1). The initial pelvis exam includes urethral, perineal, rectal, and vaginal exams as well as a general assessment of mechanical stability (6). Mechanical stability is assessed by manual compression of the iliac wings and/or greater trochanters. Distraction should be avoided due to the possibility of increasing pelvic volume and allowing further bleeding (1). Pelvic compression should be done one time only, by a senior member of the trauma team, to limit the risk of disrupting an existing blood clot (1). Other physical exam findings that suggest pelvic fracture include evidence of ruptured urethra (scrotal hematoma or blood at the urethral meatus), discrepancy in limb length, and rotational deformity of a leg without obvious lower extremity fracture. At the conclusion of the primary survey, it is imperative to cover the patient with warmed blankets to help prevent hypothermia. Laboratory-tests are typically obtained during the primary survey. Sensitive laboratory markers of acute traumatic hemorrhage include serum lactate, base deficit, and gastric intramucosal pH, which have been shown to be more reliable indicators of HI than hemoglobin level and hematocrit (7, 8).

### Imaging and Injury Classification

A standard anteroposterior (AP) pelvic x-ray (PXR) as part of the ATLS protocol can help to quickly identify life-threatening PRIs and guide triage (9–12). Chest bleeding is assessed using a chest x-ray (CXR) and abdominal bleeding with a focused assessment with sonography for trauma (FAST) exam of the abdomen (1). Classification of pelvic fractures can be accurately done from the PXR alone (13). The two most common classifications used for pelvic fractures are the Tile classification (14) and the Young and Burgess classification (15). A more recent scheme was proposed in 2018 by the AO/OTA (16). The Tile classification is focused on biomechanical stability of the sacroiliac complex (14), whereas the Young and Burgess classification is focused on mechanism of injury and has also been associated with degree of blood loss (15). Lateral compression mechanism (LC) fractures with increasing involvement, 1–3, exhibit an increased incidence of pelvic vascular injury, retroperitoneal hematoma, shock, and 24-h volume needs (17). Anteroposterior compression (APC), types 1–3, exhibit an increased incidence of injury to spleen, liver, bowel, pelvic vascular injury with retroperitoneal hematoma, shock, sepsis, and ARDS, and large increases in volume needs, with incidence of brain and lung injuries in all grades. The pattern of injury in APC3 was correlated with the greatest 24-h fluid requirements and with a rise in mortality as the APC grade rose (17). The vertical sheer (VS) mechanism, despite being highly unstable mechanically, has more recently been shown to have very low transfusion requirements (18–22). A suggested explanation is that fracture types such as VS and LC1 involve shortening of the vascular structures (21, 22), whereas LC3, APC2, and APC3 fractures involve stretching and tearing of the veins and arteries adjacent to the posterior pelvic ring (21–23). Fracture displacement in proximity to major arteries are associated with the highest risk for arterial injury (24). These locations include vertically displaced fractures in the middle part of the superior pubic ramus, the area along the internal surface of the ischial ramus, and the inferior pubic ramus, as well as in the apex of the greater sciatic notch and around the ventral part of the sacroiliac joint (24). A pelvic computed tomographic (CT) scan can allow more precise classification as well as assessment of the size of a pelvic fracture hematoma (25). CT hemorrhage volumes exceeding 500 ml have a 45% rate of pelvic arterial injury compared to 5% in volumes below 200 ml (26). A pelvic hematoma of >3.35 cm in size is correlated to an increased need for angiography and increased mortality (27). The presence of intravenous contrast extravasation on a CT scan, often called a “blush,” indicates vascular disruption and active arterial bleeding (either contained or free) (28–30). A triple-phase contrast-enhanced CT consists of an arterial phase, a portal phase, and a delayed phase (31, 32). A blush on the arterial phase indicates active arterial bleeding; it can be seen as a hyperdensity within a hematoma. A delayed phase may show injuries to urologic structures. Presence of a blush is not an absolute indication for an operative or angiographic intervention (28, 29), and because of hypotension or arterial spasm, in the absence of a blush it cannot be assumed that there is no active bleeding (30). Furthermore, bleeding from the marrow of the fractured bone can lead to significant HI (33).

### The Pelvic Binder

A pelvic binder (PB) is a device used to compress the fractured pelvis in an effort to stop bleeding (1). PBs can improve hemodynamic stability and therefore should be applied as soon as the patient with HI is suspected of having a PRI. The PB can be applied when first seeing the patient in the field or in the ambulance as pelvic bleeding can cause HI very rapidly. Use of PBs is associated with significant reduction in transfusion requirements (34, 35), as well as shorter intensive care duration and shorter hospital stay (36). There are no contraindications to applying a PB on a suspected PRI. The sheet or binder is applied at the level of the greater trochanters, never around the abdomen or waist, and should be flat against the skin to maximize surface area. Sheets should be secured with clamps to avoid undue pressure from knots (37, 38). An appropriately placed binder and sheet should allow for groin or abdominal access as holes can be cut out to provide access (34, 39). The best results of external pelvic stabilization are achieved in rotationally unstable LC fracture type III and APC types II and III (40, 41). With additional vertical instability (VS or CM type injury), stabilization can be achieved in only 27% of cases and supplementary ipsilateral skeletal traction is needed (40, 41). There have been concerns that a pelvic binder may produce secondary displacement of a lateral compression (LC) fracture causing further damage. A small increase in internal rotation and reduction of pelvic inlet area in unstable LC type II fracture has been reported with the application of a binder in cadaveric studies (42). However, there are no case reports in the literature of binder application causing damage or significant displacement in lateral compression injuries (43). Furthermore, the degree of displacement of the pelvic ring is likely to be far greater at the time of injury than afterwards with the application of a binder. Therefore, PB can be used in all LC fractures (43). Pelvic binder use should be limited to 24–48 h. Risk of complications such as skin necrosis and pressure ulcerations increase by continuous application of a pressure above 9.3 kPa for more than 2–3 h (44).

### Triage

**Figure 1:**
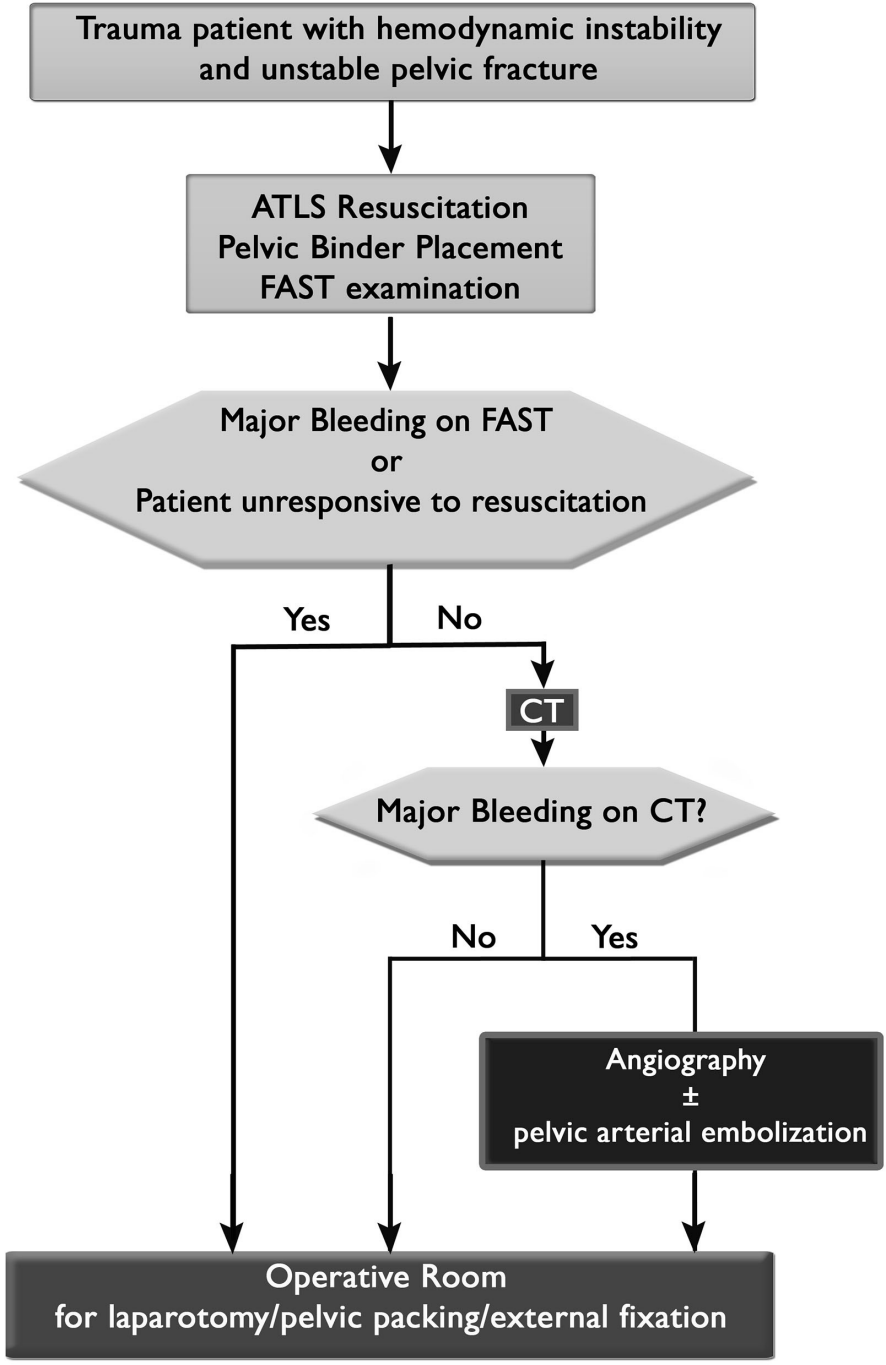
Triage algorithm for patients with hemodynamic instability and unstable pelvic ring injuries.

Diagnostic workup strategies in the emergency room must be standardized and streamlined in order to avoid an unnecessary delay to definitive bleeding control (45). Provided that the patient has HI and a mechanically unstable pelvis on PXR, the first triage decision is whether a patient is stable enough to go to the CT scanner (Figure 1). As soon as the patient is hemodynamic stable then CT of abdomen and pelvis with contrast should be used in order to detect major bleeding. The second triage decision is whether to take the patient to the operating room, the angiography suite, or the intensive care unit. If the patient is responsive to resuscitation and has evidence of arterial bleeding on CT then angiography and arterial embolization for hemodynamic stability are the next step, followed by mechanical stabilization (either internal fixation or external fixation) of the pelvis in the operating room (OR). If the patient has HI and is unresponsive to resuscitation efforts then the patient should undergo a FAST exam in the trauma bay and then go to the OR for laparotomy (positive FAST) or mechanical stabilization and pelvic packing (negative FAST or retroperitoneal bleeding identified during laparotomy). This is then followed by CT scan and either angiography or ICU based on CT results (46). A subject of more controversy is where to send the patient that has HI and a mechanically unstable pelvis but does not have arterial bleeding on CT or has evidence of arterial bleeding but is responsive to initial resuscitation. Proponents of angiography and embolization first (47) or mechanical stabilization first (48) exist. Regardless, resuscitation efforts should continue, uninterrupted, in these patients.

## Resuscitation

The initial management of the patients with hemodynamically unstable PRIs revolves around patient resuscitation. Over the past few decades there has been a greater appreciation for the physiologic disequilibrium that follows acute trauma. The classic “lethal triad” included a combination of hypothermia, coagulopathy, and acidosis, each of which furthered a patient's physiology away from normal and are associated with poor outcomes (49–51). Over the past decades, a “two hits” model of systemic inflammatory response in the critically injured patient has been described (52). The initial trauma acted as the first hit that leads to the initiation of an immune–inflammatory response with potential interventions done during this period such as surgical interventions being a potential “second hit” driving a patient toward a more systemic inflammatory response characterized by organ dysfunction and is a cause for later morbidity and mortality following acute trauma. The immunological activation and response to inflammation have a genetic background. An intervention done during this period such as surgical interventions consist a potential “second hit” driving a patient toward a more systemic inflammatory response characterized by organ dysfunction and is a cause for later morbidity and mortality following acute trauma. The concept of the second hit has now been superseded by the concept of the “genomic storm” in which up to 80% of the leukocyte transcriptome is altered together with suppression of genes involved in adaptive immunity (53). Efforts to prevent this so-called “second hit” has led to the development of damage control resuscitation (52).

Damage control resuscitation refers to an overall staged approach to care for the critically or multiply injured patients rather than a specific intervention (52, 54). Damage control resuscitation was initially described in penetrating abdominal trauma but the concept was subsequently extended to skeletal trauma as well including PRIs (52, 55, 56). The primary goal of damage control is perform life-saving interventions while minimizing the initial surgical burden to allow for restoration of a patient's physiology so as to minimize the burden of the “second hit.” Thus, the initial focus is on treating blood loss, addressing coagulopathy, and correcting acidosis. Early studies that demonstrated pulmonary and systemic benefits by early definitive fixation of femur fractures led to the concept of “Early Total Care.” The increased understanding of the physiology of the trauma patient and the need to avoid the “second hit” led to DCS. Most recently, the concept of Early Appropriate Care (EAC) has been introduced. With EAC the patients are taken to definitive fixation of their fractures as soon as they are considered resuscitated by physiological parameters and laboratory findings (57). Identification and temporizing measures for PRI associated blood loss are discussed in other sections of this paper.

In terms of restoring blood volume, high volume crystalloid solutions were historically used for fluid resuscitation. In exsanguinating patients, aggressive, and early resuscitation with blood products has been associated with increased survival rates (58–61). The increased recognition of the role of coagulopathy in disrupting a patient's physiology and worsening outcomes raises concerns that crystalloid based resuscitation contributes through dilutional coagulopathy and possibly via the disruption of pre-mature blood clots in areas of injury leading to further blood loss (50, 51). Given these concerns there has been an increasing adoption of early blood product administration. While management of coagulopathy was historically an afterthought after a patient received large blood volumes, there is increased recognition of the benefit of transfusing mixtures of packed red blood cells, platelets, and fresh frozen plasma. Though there has been much written on the optimal ratios of products to use in specific scenarios and patient populations, early product transfusion is now the mainstay for the treatment of acute traumatic blood loss and coagulopathy (49, 60, 62, 63). Further, permissive hypotension has been advocated for resuscitating patients such that end organ perfusion is maintained while minimizing hydrostatic clot disruption with a goal SBP between 80 and 90 mmHg (52, 54). Vasopressors are typically reserved either only when SBP goals cannot be maintained despite the initial fluid expansion or if there are mean arterial pressure goals such as maintaining cerebral perfusion pressure in patients with head injuries or in the case of spinal cord injuries (64).

Coagulopathy is associated with increased bleeding, organ dysfunction, and mortality and must be addressed (49, 65–67). This is secondary to clotting component consumption, hemodilution, component loss in the setting of hemorrhage, and component dysfunction in the setting of acidosis and hypothermia. Recognition of this has driven the increasing incorporation of factor transfusion in the resuscitative process. In addition to the common measures of coagulopathy (e.g., PT and PTT), there have been recent advances in laboratory medicine such as thromboelastography or rotational thromboelastometry which seek to identify whether a coagulopathy can be attributed to platelet function, clotting strength, or fibrinolysis and thereby guide treatment in polytrauma patients with PRIs (68–71). However, such methods are not yet widely available (54).

With regards to acidosis correction, the mainstay is to address the above issues by addressing ongoing blood loss and end organ perfusion (52, 72). Pharmacologic agents to correct acidosis have not been shown to offer much advantage (72). However, measures of acidosis such as a lactate or base deficit are very useful as a measure of a patient's overall response to resuscitative measures.

## Pelvic Arterial Embolization and Pelvic Packing

After initial assessment, binder placement, and resuscitation, hemodynamic status is reassessed to determine whether the patient is responding to treatment. If hypotension persists, the decision must then be made of whether the patient is sufficiently stable for angiographic embolization in the interventional radiology suite or pelvic packing in the operating room.

It has been estimated by (73) in a study of 27 post-mortem angiographies that only 11.1% of patients with pelvic fractures exhibit arterial bleeding. Otherwise, bleeding is primarily attributed to veins and fractured cancellous edges. The ideal indication for angiography is arterial hemorrhage, yet discerning the source of hemorrhage is not a simple task. In a prospective multi-center observational study of 1,339 patients with pelvic fractures conducted by the American Association for the Surgery of Trauma, Costantini et al. (74) reported little consensus among 11 level one trauma centers regarding management of hemorrhage in the setting of pelvic fracture. Their cohort included 178 patients admitted in shock (defined as SBP < 90 mmHg, HR > 120 bpm, or base deficit > −5), of which 18.5% were initially treated with pelvic binder, 24.7% went for diagnostic angiography (68.2% of whom were embolized), 9.6% were treated in an external fixator alone, 5.1% underwent pre-peritoneal packing alone, 5.1% underwent external fixator plus embolization, 1.7% external fixator plus pre-peritoneal packing, and 68% no pelvic intervention. Mortality in this series was 32% for patients with pelvic fracture presenting in shock (74). In a nationwide survey of 40 trauma medical directors in the US, 33% perceived pelvic packing as effective, 72% perceived pelvic packing as safe, and 76% reported that packing was utilized as the third or fourth priority in their treatment algorithm. Geographic trends were seen with regard to utilization (75).

### Angiographic Embolization

The first published report of pelvic angiography in the setting of pelvic fractures was in a 1971 editorial by Athanasoulis et al. (76) from Boston City Hospital, commenting on the possible utility of initial venography and subsequent arteriography as a diagnostic tool to identify the location of bleeding in the pelvis. A small series of therapeutic embolization was published 1 year later by Margolies et al. (77) at Massachusetts General Hospital, who selectively catheterized a branch of the internal iliac artery to localize the site of bleeding, followed by embolization using autologous clotted blood. This was a particularly attractive option given the alternatives at the time, which included ligation of the internal iliac artery, a practice met with little success given the large number of collateral vessels in the pelvis (78).

The common iliac arteries bifurcate from the aorta around the L4 spinal level, continuing into the pelvis before branching into the internal and external iliac arteries. The external iliac arteries continue over the pelvic brim as the common femoral arteries, and the internal iliac arteries supply the majority of the pelvis via multiple named branches. These include the superior gluteal, inferior gluteal, iliolumbar, lateral sacral, umbilical, obturator, internal pudendal, middle rectal, uterine (in females), and superior vesical branches. Embolization can be selective, which targets a specific branch of the internal iliac artery, or non-selective, which plugs the arterial leak upstream at the internal iliac artery. Selective embolization is preferred when possible, though the indication to non-selectively embolize may be appropriate in the setting of a hemodynamically unstable patient. The vessels most commonly embolized are, in descending frequency, the internal iliac (67.2%), unnamed branches of the internal iliac (17%), superior gluteal artery (4.4%), obturator artery (4.1%), and internal pudendal artery (3.2%) (79). Embolization can be executed with various media, including coils, gelfoam pledgets, vascular plugs, particles, liquid embolics, or a combination of these (80), with coils and gelfoam being the most commonly employed in the authors' experience. Coils work by inducing thrombosis, often enhanced by Dacron wool tails, not mechanical occlusion. They are permanent devices and are effective in proximal embolization. Gelfoam is a water soluble temporary embolic agent that is completely resorbed within 2–3 weeks (80).

Time from injury to intervention has been shown to make a notable difference in outcome. Agolini et al. (81) reviewed 806 patients with pelvic fractures from a single institution and found that 4.3% of them underwent angiography, and 1.9% underwent embolization. Patients embolized within 3 h had a mortality rate of 14%, compared to 75% in those embolized after 3 h (81). Tanizaki et al. (74) analyzed 140 patients with pelvic fractures, 24 of which were hemodynamically unstable. Patients who underwent embolization within 60 min of arrival had a mortality rate of 16% compared to 64% in those over 60 min (47).

Repeat embolization may be required in select patients. Fang et al. (82) reviewed 140 patients with pelvic fractures who underwent embolization for suspected arterial hemorrhage, 19% of which required repeat embolization. Predictors for repeat embolization in their series were initial hemoglobin <7.5, superselective embolization at the time of the initial procedure, and >6 units of transfused red blood cells after initial embolization. The source of bleeding occurred at a new site in 38% of patients. The authors recommended maintaining the sheath in the femoral artery for 72 h should the need for repeat angiography and embolization arise (82).

Complications of diagnostic angiography include contrast mediated kidney injury, allergic reaction, and cannulation site morbidity. Complications of embolization include subsequent surgical wound breakdown, deep infection, gluteal muscle necrosis, nerve injury, bladder or ureteral infarction, bowel infarction, claudication, and impotence. Reported complication rates range from 3 to 35%, though the deleterious impact of the traumatic event itself is likely a confounding variable (79, 83–85). Non-selective embolization of the internal iliac artery, particularly bilaterally, may lead to higher complication rates (84), though this point has been contested in the literature (86, 87). Such a technique should be considered with caution, though hemodynamic instability in select settings may be an appropriate indication.

Mortality rates are likewise multifactorial, making reports of angioembolization difficult to interpret. Among patients with pelvic fractures presenting in shock treated primarily with angioembolization, mortality rates range from 12 to 40% (83, 87–93).

### Pelvic Packing

Vague reports make it difficult to discern the origin of pelvic packing, though mention is made by Quinby in a 1971 editorial, alluding to its inefficacy in a mechanically unstable PRI because “there is little in the way of stable platforms to pack against” (94). The first complete description of packing was by Pohlemann and Tscherne in Hannover Germany in a technique paper published in 1995 (95).

Drawing from their observations in Europe, Cothren et al. published a technique in 2007 shown to be effective in a large series. Making a 6–8 cm vertical skin incision starting just above the pubic symphysis, dissection is taken carefully through the rectus sheath. The fracture hematoma often dissects the pre-peritoneal space (of Retzius) beforehand, but this can be done bluntly with a finger. Blunt dissection continues in the paravesical space back to the presacral region, and three laparotomy sponges are placed. The same steps are repeated for the contralateral side. Closure includes reapproximation of the rectus sheath with 0-PDS suture and skin staples. If a laparotomy is performed, it may be done so through a separate incision placed cranially to separate the two wounds. An added benefit of this technique is that multiple teams can work concurrently (96, 97).

The abdominopelvic cavity wall is made up of seven distinct tissue planes, forming a histological palindrome. From superficial to deep, they include the skin, subcutaneous fatty tissue, external muscle fascia, muscle layer(s), internal muscle fascia, extraperitoneal fatty tissue, and peritoneum. The abdominopelvic viscera, including the bladder, kidneys, ureters, and major vessels, are found within the extraperitoneal fatty layer, and it is primarily this expansive layer that fills with blood during pelvic fractures, and this selfsame layer into which packs are placed.

Intuitively, similar to angioembolization, timing of pelvic packing is thought to play a significant role in outcomes, though comparative studies are lacking. Burlew et al. (97) reported their mean time from admission to the operating room as 44 min with 21% mortality rates. The authors are not aware of a series comparing outcomes as a function of time (97).

Burlew et al. have published one of largest recent series utilizing a pre-peritoneal packing protocol over 11 years, including 128 patients with pelvic fractures presenting with persistent SBP < 90 mmHg after placement of a binder or sheet and 2 units of pRBC. Mean ISS was 48 and 18 of the fractures were open. Twenty-seven percent of patients underwent diagnostic angiography after pre-peritoneal packing given concern for continued bleeding, 46% of whom went on to embolization. Twelve percent of patients in this series experienced pelvic space infection, and the mortality rate was 21% (97).

Few comparative studies have been published of angioembolization and pelvic packing. In a retrospective single-institution series of 40 hemodynamically unstable patients with pelvic fractures, Osborn et al. (98) compared 20 patients who underwent pre-peritoneal packing (mean time from admission 45 min) with 20 patients who underwent angioembolization (mean time from admission 130 min). In those who underwent pre-peritoneal packing, four died, none of which due to hemorrhage, compared to six deaths in the angioembolization group, two from acute hemorrhage (98).

Li et al. (99) conducted a quasi-randomized controlled trial of hemodynamically unstable PRI patients, 29 of whom were randomized into a pelvic packing group, and 27 of whom were randomized into an angiography group. Of those who underwent packing, ISS was 48, time to surgery was 77 min, operative time was 60 min, and 4 patients died (none from hemorrhage). Of those who underwent angiography, ISS was 43, time to intervention was 102 min, procedure time was 84 min, and 5 patients died (two from exsanguination) (98, 99).

## External Fixation

In patients with hemodynamic instability following PRI, mechanically stabilizing the osseous structures and reducing pelvic volume are important means to prevent further bleeding. In the emergency setting, these goals need to be achieved without excessive technical complexity or time-consuming interventions.

The pelvic binder remains the default non-invasive method for rapid temporary stabilization of the pelvis. In fact, when compared to an external fixator, there were no significant differences in stability conferred by an external fixator vs. a pelvic binder for unstable pelvic injuries (100). The shortcoming of a pelvic binder are soft tissue complications secondary to the pressure that is applied (34, 37, 101), in addition to reduced access to the abdomen, pelvis, and groin areas, as well as decreased ability to deal with associated injuries involving the hip and lower extremities. Additionally, a binder is temporary and not a means for definitive treatment of a PRI.

These issues are overcome by external fixation of the pelvis, which provides mechanical stability and can serve as a temporizing measure or as definitive treatment. The efficacy of early application of external fixators has been previously demonstrated, with endpoints of decreased morbidity, decreased transfusion requirements, and improved mortality (19, 95, 102, 103). External fixators are applied in a percutaneous manner and function well to provide fracture stabilization, tamponade expanding hematoma, and reduce hemorrhage from fracture sites. In patients that undergo pre-peritoneal pelvic packing, application of external fixation is necessary as a means to provide counter pressure for effective pelvic packing (98, 104, 105). External fixation should also ideally precede laparotomy as a means to prevent the disruption of the tension band created by the abdominal wall on the iliac wings (103, 106).

The type of PRI dictates the appropriate external fixation construct. Other considerations are patient body habitus, available imaging, and surgeon experience (107). Anterior external fixation constructs are most appropriate for APC and LC-type injuries and work particularly well when the posterior ligaments are intact (107). The anterior external fixator can be placed with pins in the iliac crests, in the supra-acetabular region, or less commonly, in the subcristal area. Placement of pins in the iliac crests can be done in the emergency department, with or without fluoroscopy, and is likely the fastest option. Biomechanically, supra acetabular pins are advantageous due to stronger bone in the sciatic buttress and a better vector for pelvic ring closure (40, 107). In both constructs, when correctly assembled, access to the abdomen can be unimpeded (108). Disadvantages of external fixation include poor patient tolerance, pin site infection, and aseptic loosening (109).

In posterior pelvic ring disruptions, an anteriorly based fixator is ineffective in delivering posterior compression (110). This can be improved by using a femoral distractor as a compressor or by modifying the anterior frame into an X configuration (X frame) (110, 111). Nonetheless, in injuries involving significant posterior diastasis, the posteriorly based pelvic C clamp is more effective (112, 113). The C clamp consists of two sidearms connected by a crossbar and is meant to be anchored in the posterior ilium, a few centimeters anterior to the PSIS (114). Contraindications to its use include comminuted or transforaminal sacral fractures, iliac wing fractures, and LC-type fractures (32, 115). Theoretically, the C clamp can be applied in the emergency department without fluoroscopy, however, it is advantageous to apply it in the operating room under fluoroscopic control, given that major complications, such as fracture displacement, perforation through the ileum, nerve injury, and hematoma formation can arise from inadvertent misplacement (116–118). An advantage of the C clamp is that it can rotate around its axis, allowing access to the abdomen and groin. The C clamp can also be used for anterior pelvic disruption, such as APC 2, when it is applied anteriorly. External fixation, either with formal half pins or by some form of temporary pelvic C-clamp, can be useful in providing pelvic stability (34), however, both devices provide limited control of the posterior pelvis. Richard and Tornetta (119) and Archdeacon and Hiratzka (120) described an alternative technique involving application of a C clamp at the greater trochanters (T-clamp). In a recent case series of 17 hemodynamically unstable patients requiring pelvic stabilization, the T-clamp was a safe and effective method for pelvic stabilization (121).

## Internal Fixation

The continuum of management of hemodynamically unstable patients with PRI ranges from damage control to early total care. In the acute setting, when dealing with a patient with HI and an unstable pelvic fracture, most authors agree that proceeding with definitive fixation of the pelvic fracture is not advocated due to the time consuming nature of the procedure and inherent risk from extensile approaches, pre-disposing the patient to increased hemorrhage, acidosis, hypothermia, and coagulation disturbances (122, 123).

Nevertheless, there is some evidence that maximizing early fixation of PRIs may be advantageous, or at least, not deleterious to patients. In a review of 45 patients with unstable pelvic fractures, Enninghorst et al. (124) found that despite having initial worse shock parameters, the acute ORIF group did not differ from the staged ORIF group with respect to transfusion rates, mortality, ICU, and overall length of stay, concluding that acute internal fixation could be performed even in critically ill patients (124). Further support for timely intervention is provided by Vallier et al. (125) who found a complication rate of 12.4 vs. 19.7% in patients treated in <24 and >24 h from injury, respectively, including less ARDS and pneumonia.

The anterior external fixator, as discussed, is an excellent tool for closing down the anterior aspect of the pelvic ring. An alternative to the anterior external fixator is the anterior subcutaneous internal fixator (INFIX), which consists of a metal rod passed subcutaneously and connected to two pedicle screws anchored in supra acetabular bone (126). It attempts to address the issues of pin tract infections, osteomyelitis, loosening, loss of reduction, difficulty of use in obese patients, and restriction of movement (126). However, complications of the technique include lateral femoral cutaneous nerve irritation, femoral nerve palsy, and heterotopic ossification (127, 128). While the INFIX has a role, it is likely less appropriate in the emergency setting, where a percutaneously applied anterior external fixator can rapidly and effectively reduce the anterior pelvic ring.

Due to concerns regarding the use of a C-clamp (116–118), alternative methods of posterior ring fixation have been devised. In trained hands, acute percutaneous stabilization of the posterior pelvic ring is possible through the use of an iliosacral screw (anti-shock screw) (129). Familiarity with the technique and ability to execute rapidly is required. The risks of the technique include misplacement of the screw causing neurologic or vascular injury, inaccurate reduction, and mechanical failure of fixation (130). In cases where reduction is suboptimal, revision of fixation is possible once the physiologic status of the patient permits.

## Special Considerations

### Pediatric Pelvic Ring Injuries

In general, pediatric PRIs fall under two camps, low energy pelvic avulsion fractures caused by lower energy mechanism such as during sports, and higher energy pelvic ring or acetabular disruptions (131). The skeletally immature pelvis is characterized by a greater portion of cartilaginous structures with variable rates of ossification. This factor, combined with flexibility through the tri-radiate cartilage and thicker periosteum lends itself to the greater elasticity of the pediatric pelvic ring which is thought to account for the lower incidence of PRIs in pediatric patients as compared to adult patients. However, when a ring disruption is present, it is often due to a high energy mechanism and suspicion must be raised for associated face/head (49–58% of patients), thoracic (7–31%), extremity (47–62%), and abdominal (25%) injuries (132–136). In terms of resuscitation of pediatric patients, the vasculature in children is more reactive and as such, early stages of hemorrhagic shock can be masked until patients are in the late stage. The initial evaluation is guided by the advanced pediatric life support protocols. Though the need for blood transfusion and angiographic intervention is similar between children and adults (131), the overall mortality in patients with pelvic fractures has been shown to be lower in children as compared to adults (5 vs. 17%) with pelvic fracture associated hemorrhage occurring far less frequently in the pediatric population (137).

### Pelvic Ring Injuries During Pregnancy

Despite the low prevalence of pregnancy amongst patients presenting with PRIs (0.2–1.1%), (138, 139) patients require immediate multidisciplinary care between obstetrics, general trauma surgeons, orthopedic traumatologists, anesthesia, and maternal-fetal medicine providers (140). Though maternal mortality in trauma is similar to mortality amongst non-pregnant patients, fetal mortality can be as high as 50–65% in the setting of severe blunt trauma and correlates with elevated injury severity score, maternal hemorrhage, coagulopathy, and abruptio placentae (140–142). Specifically with regard to hemodynamic instability, hypovolemic shock as with hemorrhage can decrease placental blood flow up to 20% contributing to higher rates of fetal demise (143). On initial evaluation, the primary goal remains restoration of the mother's physiology with resuscitative efforts, prompt diagnosis and initial treatment. The presence of vaginal blood must be evaluated for the presence of an open fracture, placenta previa, placental abruption, or labor (140). If a pelvic binder is to be used, it must not compress the gravid uterus and thereby lead to compression of the inferior vena cava. The resulting cardiac physiology changes associated with pregnancy, namely the increased plasma volume, may mask early signs of hypovolemic shock. Blood products must be Rh negative if the mother's blood type is unknown and a Kleiheir-Betke blood-test or anti-HbF flow cytometry for circulating fetal hemoglobin may be considered to detect maternal-fetal hemorrhage (144, 145). Further, logrolling to the left lateral decubitus position is preferential to the straight supine position, especially later in pregnancy (140).

### Geriatric Patients

The incidence of pelvic fractures has been shown to be higher in geriatric populations (146, 147). Geriatric patients have less physiologic reserve to respond to the stress of trauma, are more susceptible to clinically significant injuries, and have higher mortality, even in the setting of low energy mechanisms (148). Under triage remains a concern as up to 63% of patients with injury severity scores >15 have been shown to not meet the standard hemodynamic criteria for HI such as SBP <90 mmHg or heart rates above 120 bpm (149). Systemic vascular resistance is often increased in the setting of baseline hypertension and heart rates may be blunted either due to ischemic heart conditions, a decreased response to catecholamines, or medications such as beta blockers. As such, modified criteria such as including age alone, SBP <110 mmHg, and heart rate >90 bpm have been suggested as triaging criteria for elderly patients (149). Providers must maintain a high suspicion for shock in this patient cohort. Further, the presence of comorbid conditions such as heart failure or renal disease must be considered during the resuscitation as fluid overload may cause further morbidity. Given the above, serum lactate and base deficit may be better markers of resuscitation and end organ perfusion than vital signs in this patient population. With regards to PRIs and HI specifically, despite sustaining less severe fracture patterns, geriatric patients required greater rates of transfusion, undergo interventional angiography, and die despite aggressive resuscitation (150, 151). The increased bleeding in older patients has been postulated to be related to decreased vessel compliance seen with age and the decreased ability for vasospasm. In the setting of HI, the presence of anticoagulants must be addressed with consideration given to anticoagulation reversal (152). The pre-injury anticoagulant and antiplatelet therapy is related with increased risk of post-traumatic hemorrhage (152, 153). Additionally, on an observational study about hip fractures pre-operative use of antiplatelet drugs was associated with an increased risk of transfusion and mortality and the use of non-vitamin K antagonist oral anticoagulant was associated with an increased risk of blood transfusion (154).

## Conclusions

Pelvic ring injuries associated with hemodynamic instability require an expeditious evidence based and protocol driven approach to management. A pelvic binder should be applied as soon as hemodynamic instability is suspected followed by a thorough physical exam, a pelvic x-ray and in most cases a CT scan. Triage decisions are largely based on the patient's response to resuscitation and the results of imaging studies. The choice of taking the patient to the invasive radiology suite for pelvic artery embolization, the operating room for pelvic external fixation and possibly packing, or the intensive care unit should be the product of evidence-based protocols, surgeon experience, and institutional availability of the specific resources. Resuscitation efforts should consider specific patient populations and continue uninterrupted regardless of treatment or triage and until the patient is hemodynamically stable. Future research is needed to further identify the causes of hemodynamic instability in the trauma patient, an particularly the patient's response to resuscitation and the optimal timing of surgical intervention.

## Author Contributions

All authors participated in the literature search, writing, and editing of the manuscript.

## Conflict of Interest

The authors declare that the research was conducted in the absence of any commercial or financial relationships that could be construed as a potential conflict of interest.
